# Pathophysiology and risk factors for postoperative nausea and vomiting in adults and children

**DOI:** 10.1016/j.bjae.2025.02.003

**Published:** 2025-04-16

**Authors:** A.L. Kovac

**Affiliations:** University of Kansas Medical Center, Kansas City, KS, USA

**Keywords:** anaesthesia, postoperative complications, postoperative nausea and vomiting

**Learning objectives**

By reading this article, you should be able to:

• Discuss the aetiology and pathophysiology of postoperative nausea and vomiting (PONV), postoperative vomiting (POV) and postdischarge nausea and vomiting (PDNV) in adults and children.
• Describe the risk factors related to the patient, surgery and anaesthesia for PONV, POV and PDNV in adults and children.
• Compare how PONV differs between adults and children.
• State the PONV risk scoring systems available for use in adults and children.

**Key points**

• The causes and incidences of postoperative nausea and vomiting (PONV), postdischarge nausea and vomiting (PDNV), and postoperative vomiting (POV) can be different between adults and children.
• Although specific surgery and patient-related risk factors cannot often be changed, anaesthesia and medication-related factors can be minimised, removed, or both.
• The perioperative use of nitrous oxide, volatile anaesthetics agents and opioids are some of the most important risk factors for PONV.
• Risk assessments and scoring methods can help decrease PONV in adults and children.

Postoperative nausea and vomiting (PONV) can affect patients' satisfaction, medical costs, adverse events, morbidity and mortality in adults and children undergoing surgery and anaesthesia. The proper approach to prevent and treat PONV is guided by an understanding of the pathophysiology, stimuli and mechanisms that affect risk for PONV.

Table 1 describes the connection between the central nervous system (CNS) and the resulting physiological effects. The CNS areas and receptors involved in nausea and vomiting are shown in Fig 1, Fig 2. These include the vomiting centre, fourth ventricle, area postrema, chemoreceptor trigger zone (CTZ) and nucleus of the tractus solitarius. When receptors in the peripheral gastrointestinal (GI) tract, inner ear and vestibular system are stimulated, nausea, retching and vomiting occur. Controversy exists as to whether the sequence of autonomic output events, such as pallor, salivation, sweating and hypotension, are controlled by a localised integrative site (vomiting centre) or a localised integrative central pattern generator.^1^

**Table 1:** Central nervous system neural pathways, integration and physiological effects. CTZ, chemoreceptor trigger zone. Reproduced with permission from Stoops S, Kovac AL. New insights into the pathophysiology and risk factors for PONV. *Best Pract Res Clin Anaesthesiol* 2020; **34**: 667–79.

Brain cns neural pathways, integration, and physiology	
CNS pathways	• Brain, cerebral • Inner ear, vestibular system • Vomiting centre, area postrema, CTZ • Abdominal vagal afferents • Serotonin release from enterochromaffin cells of duodenum • Nucleus tractus solitarius
CNS input and integration	• Pons • Cerebellum • Ventral lateral medulla • Salivary nuclei • Dorsal motor nucleus • Retrofacial nucleus • Respiratory centre
Physiological effects	• Prodromal signs, symptoms, and effects • Muscle sequence for retching • Muscle sequence for vomiting

**Fig 1:**
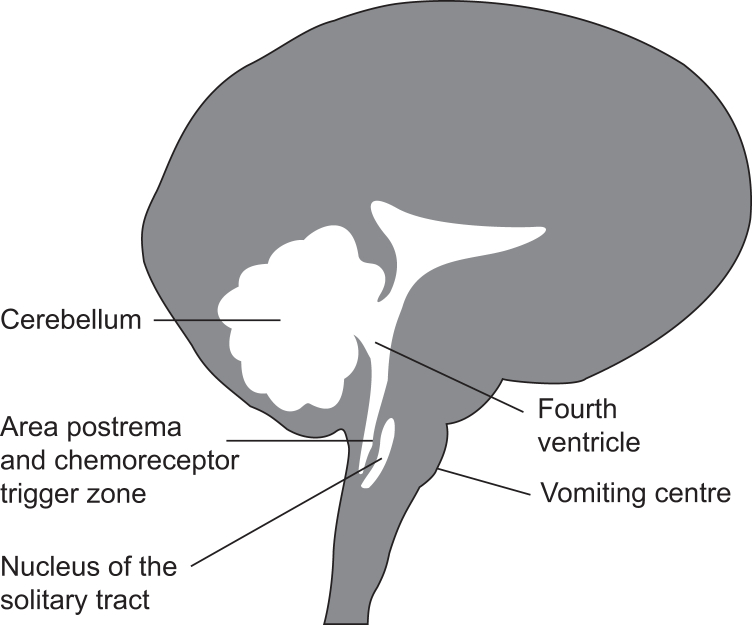
Central nervous system brain areas involved with nausea and vomiting. Reproduced with permission from Stoops S, Kovac AL. New insights into the pathophysiology and risk factors for PONV. *Best Pract Res Clin Anaesthesiol* 2020; **34**: 667–79.

**Fig 2:**
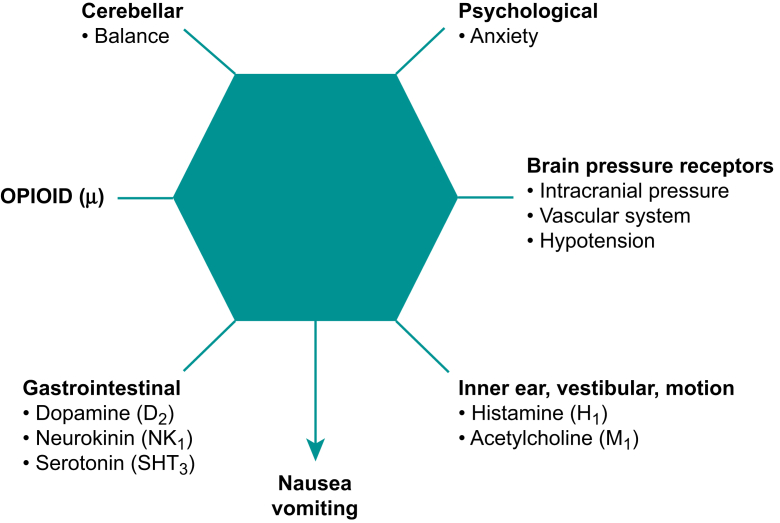
Receptors and stimuli in peripheral and central locations associated with nausea and vomiting. Reproduced with permission from Stoops S, Kovac AL. New insights into the pathophysiology and risk factors for PONV. *Best Pract Res Clin Anaesthesiol* 2020; **34**: 667–79.

A variety of receptors and stimuli that cause PONV are located in the peripheral nervous system and CNS. Given the large number of receptors and triggers involved in the genesis of PONV, a multimodal approach for prophylaxis and treatment should be considered. Opioid (μ) receptors are present in the peripheral GI tract and CNS. Histamine (H_1_) and cholinergic muscarinic (M_1_) receptors are present in the inner ear and vestibular systems. The brain CNS is the location for the vomiting centre, area postrema, CTZ and nucleus of the solitary tract. The 5-hydroxyl tryptamine-3 (5-HT_3_), dopamine (D_2_), opioid (μ), cholinergic (M_1_) and neurokinin (NK_1_) receptors are present in the brain CNS areas of the vomiting centre, area postrema and CTZ. Serotonin (5-HT_3_), neurokinin (NK_1_) and opioid (μ) receptors are in the area of the nucleus of the solitary tract.^2^

To achieve maximum benefit from prophylaxis, the patient's risk for PONV, postoperative vomiting (POV) or postdischarge nausea and vomiting (PDNV) should be evaluated. Specific risk factors in adults (Table 2) and children (Table 3) relate to: (i) type of surgery; (ii) patient's characteristics; (iii) anaesthesia; and (iv) postoperative medications that a patient may receive.^2^^,^^3^

**Table 2:** Apfel simplified postoperative nausea and vomiting (PONV) risk score for adults. The incidence of PONV increases as the total number of risk factors increases. Each additional risk factor increases PONV risk approximately 20%. Reproduced with permission from Stoops S, Kovac AL. New insights into the pathophysiology and risk factors for PONV. *Best Pract Res Clin Anaesthesiol* 2020; **34**: 667–79.

PONV risk factors	Number of risk factors	PONV risk (%)	Degree of PONV risk
• Female sex	0	10	Low
• History of PONV or motion sickness	1	21	Mild
• Non-smoker	2	39	Moderate
• Postoperative opioids	3	61	High
	4	79	Extremely high

**Table 3:** Postoperative vomiting (POV) risk score for children. PONV = postoperative nausea and vomiting. Reproduced with permission from Stoops S, Kovac AL. New insights into the pathophysiology and risk factors for PONV. *Best Pract Res Clin Anaesthesiol* 2020; **34**: 667–79.

POV risk factors	Number of risk factors	POV risk (%)
• Strabismus surgery	0	10
• Age ≥3 yrs	1	10
• Duration of surgery >30 min	2	30
• History of POV or PONV in relatives (mother, father, siblings)	3	50
	4	70

## Surgery-related factors

Surgery-related risk factors include the duration and type of surgery, and indirect mechanical effects, direct mechanical effects or both on the GI tract. In adults, the incidence of PONV increased from 2.8% to 27.7%, respectively, for surgeries lasting more than 3 h compared with those shorter than 30 min.^3^ Children have a greater risk of PONV when undergoing surgical operations lasting longer than 30 min.^4^

The PONV risk is increased after gynaecological, urological, ear, nose, throat (ENT), GI and neurosurgical surgeries. Swallowed blood is also a strong emetogenic stimulus. Operations that cause bleeding in the airway or GI tract, such as plastic, dental, oesophageal, and stomach procedures, can have an increased incidence of PONV.^5^, ^6^, ^7^, ^8^

Airway, CNS, and ENT operations can stimulate central and peripheral vomiting centre receptors and the vagus and glossopharyngeal nerves.^9^ Hypotension can occur secondary to blood loss, inadequate fluid replacement or vasodilation after spinal or epidural anaesthesia. The resulting decrease in CNS blood flow stimulates vomiting centre receptors to cause nausea and vomiting.^10^^,^^11^

Surgery on the oesophagus, stomach, abdomen, colon and specific surgeries, such as laparoscopic hysterectomy and cholecystectomy (in adults), and strabismus repair or adenotonsillectomy (in children), can be associated with a higher risk of PONV.^12^, ^13^, ^14^ Strabismus surgery can cause PONV from the surgeon's traction of the extraocular muscles. Swallowed blood can be a cause of PONV during tonsillectomy. Other paediatric surgeries associated with increased risk of PONV or POV are GI procedures and craniotomies.^2^^,^^4^

Reportedly, PONV can occur in patients undergoing laparoscopic abdominal surgery resulting from increased abdominal pressure caused by abdominal insufflation of carbon dioxide gas and the head-down Trendelenburg position.^12^, ^13^, ^14^

## Patient-related factors

Patient-related risk factors (Table 2, Table 3) include age <50 yrs, female gender, non-smokers, history of PONV, motion sickness or both, and postoperative opioids.^15^^,^^16^ Gravid women who underwent non-obstetric procedures under general anaesthesia had a similar incidence of PONV compared with non-gravid patients.^17^ The vestibular system is an important cause of PONV as history of motion sickness is a patient-related risk factor. Changes in body position and movement can stimulate histamine (H_1_) and muscarinic (M_2_) receptors in the vestibular system.^16^^,^^18^ Over time, patients with a history of smoking acclimate to the toxic compounds of tar, nicotine and other tobacco smoke carcinogens leading to a lower incidence of PONV in smokers than in non-smokers. Smoking also induces liver enzymes such as the cytochrome P450 system. This can cause faster metabolism of anaesthetic drugs with a resultant decrease in their emetogenic effects.^15^^,^^19^^,^^20^ Obesity, BMI, anxiety, preoperative fasting, intraoperative use of nasogastric tubes and supplemental oxygen have been shown to have limited or no clinical relevance as a cause for PONV. In addition, controversial causes of PONV include ASA physical status and time of menstrual cycle.^16^^,^^18^^,^^20^, ^21^, ^22^

Social determinants may lead to disparities in healthcare access, treatment and outcome. The impact and interaction of sex, race and socioeconomic factors have been recently discussed and evaluated.^23^^,^^24^ Questions have been raised about whether or not Hispanic and Black/African–American patients have been undertreated compared with White patients. Racial disparities in perioperative management can be avoided through standardisation of care.

Children have an approximately 40–45% incidence of PONV. Before puberty, there is equal chance of PONV in girls and boys older than 3 yrs. After puberty, girls have approximately three times more frequent PONV incidence than boys.^2^^,^^3^

## Anaesthesia-related factors

Anaesthesia-related risk factors that contribute to PONV in adults and children include nitrous oxide, volatile anaesthetic agents (isoflurane, sevoflurane, desflurane) and postoperative opioids.^25^ Nitrous oxide diffuses into closed air spaces such as the inner ear and bowel. The resulting increased pressure causes dizziness from central effects in the vestibular system and nausea from the visceral effects of small bowel distension in the GI tract. Volatile agents cause PONV by stimulating the release of serotonin in the GI tract, increasing vestibular sensitivity and stimulating the CTZ in the brain.

An increased incidence of PONV occurs in patients undergoing general compared with regional anaesthesia.^2^^,^^3^ Orthostatic hypotension occurring in the PACU can cause nausea and vomiting. A decrease in blood flow to the brain and cerebral perfusion pressure stimulates CNS vomiting centre receptors. Exposure to anaesthetic agents for more than 45 min increases the risk for PONV, as there is a longer time for the absorption of anaesthetic agents into the body. Regional anaesthesia can help minimise the occurrence of PONV by decreasing the need for opioids and volatile agents.^26^ Propofol has antiemetic properties. Eliminating gas and volatile agents (nitrous oxide, isoflurane, sevoflurane, desflurane) by using propofol for total intravenous anaesthesia (TIVA) can help decrease the chance for PONV.^25^^,^^27^ Propofol has been shown to have antiemetic properties and is useful to decrease the risk of PONV when given as a continuous infusion during TIVA. Propofol is believed to (i) enhance the activity of gamma-aminobutyric acid (GABA); (ii) inhibit serotonin 5-HT_3_ receptors; (iii) reduce dopaminergic activity and (4) have possible anti-inflammatory effects.

The use of neostigmine to reverse neuromuscular block reportedly increases the risk of PONV compared with using sugammadex.^28^

Exogenous opioids stimulate μ receptors in the vestibular system and CTZ causing PONV.^29^^,^^30^ Peripherally, opioids cause relaxation of the colon's longitudinal muscles decreasing GI peristalsis and motility. This can increase distension of the bowel and lead to ileus, which together can cause nausea, vomiting or both. Opioids also contribute to GI dysfunction by delaying gastric emptying and intestinal mobility, resulting in stomach bloating and constipation, respectively. They also stimulate the release of serotonin from 5-HT_3_ receptors on vagal afferent nerves.^30^^,^^31^

## Risk scores for PONV

Scoring methods in adults and children have been proposed to predict PONV, PDNV and POV (Table 3, Table 4).^4^^,^^15^^,^^16^^,^^19^^,^^32^, ^33^, ^34^ van den Bosch and colleagues proposed a relatively complicated risk score by evaluating multiple factors such as:^32^ (i) gender; (ii) history of PONV, motion sickness or both; (iii) smoking history; (iv) surgery type; (v) patient age and (vi) anaesthetic technique. A simpler scoring system was proposed by Koivuranta and colleagues who evaluated: (i) gender; (ii) history of PONV, motion sickness or both; (iii) smoking status and (d) surgery duration (≥60 min).^33^ By eliminating the duration of surgery, Apfel and colleagues proposed an even simpler score evaluating four factors: (i) gender; (ii) smoking status; (iii) history of PONV/motion sickness and (iv) use of postoperative opioids.^15^ The simplified PONV Apfel score (Table 2) is perhaps the most widely used score to estimate PONV risk in research and clinical practice.

**Table 4:** Apfel postdischarge nausea and vomiting (PDNV) risk score for adults. Reproduced with permission from Stoops S, Kovac AL. New insights into the pathophysiology and risk factors for PONV. *Best Pract Res Clin Anaesthesiol* 2020; **34**: 667–79.

Factors contributing to PDNV	Number of risk factors	PDNV risk (%)
• Female sex	0	7
• Age <50 yrs	1	20
• History of nausea/vomiting after anaesthesia	2	28
• Use of opioids in PACU	3	53
• Nausea in PACU	4	60
	5	89

Eberhart and colleagues described the postoperative vomiting in children (POVOC) score.^34^ They concluded that (i) strabismus surgery; (ii) surgery duration >30 min; (iii) age 3 yrs and (iv) previous history of POV in the child or history of PONV in parents or siblings increase the risk for PONV or POV. Depending on the presence of none, one, two, three or four risk factors, the estimated incidence of POV is 9%, 10%, 30%, 55% and 70%, respectively (Table 3).

More recently, Bourdaud and colleagues developed and validated a vomiting in the postoperative period (VPOP) risk score to predict the probability of POV in high-risk paediatric patients.^35^ Five independent risk factors were identified: (i) stratified age (>3 and <6; >6 and <13; or >13 yrs); (ii) duration of anaesthesia; (iii) surgery at risk; (iv) predisposition to PONV and (v) multiple opioid doses. Using a score ranging from 0 to 6, respective POV incidences were 5%, 6%, 13%, 21%, 36%, 48% and 52%.

Douville and colleagues evaluated a polygenic score for the prediction of PONV using a retrospective derivation and validation cohort study.^36^ Using genome-wide association, they identified genetic variants and a PONV polygenic risk score. However, use of this risk score did not result in a clinically meaningful improvement of PONV prediction when added to traditional PONV risk factors.

## Use of PONV scores in clinical practice

In medicine, it appears that risk prediction scores and models have increased over the past decade because of an increased interest in achieving more precise medical therapy and outcomes.^37^ However, applying risk prediction methods without understanding the limitations of external validity can lead to poor medical care.

The use of simplified PONV algorithms to help guide antiemetic prophylactic and treatment therapy with a goal to decrease the incidence of PONV is believed to result in better postoperative outcomes for more patients.^38^ Eberhart and Morin determined that PONV/POV risk scores for children were useful and should be used in clinical practice.^39^ However, worldwide, the implementation of PONV guidelines appears to vary.

Pierre and colleagues suggested that attention should centre on the clinical usefulness of PONV algorithms and ordering reminders.^40^ Using a simplified PONV risk score, 428 adult inpatients scheduled for throat, thyroid, breast or gynaecological surgery under general anaesthesia were classified as low, medium or high risk. Low-risk patients did not receive antiemetic prophylaxis. Medium-risk patients received volatile anaesthesia with droperidol or i.v. propofol anaesthesia without droperidol. High-risk patients received i.v. anaesthesia supplemented with dexamethasone and droperidol. Compared with the results of a previous survey conducted by the authors at their institution, the overall incidence of PONV decreased from 49.5% to 14.3%, with a reduction from 34% to 13.2% in the medium-risk group and from 64.3% to 15.5% in the high-risk group after the institution of risk-adapted prophylaxis. The authors concluded that the use of a simplified risk score-dependent prophylactic antiemetic strategy can significantly reduce the overall institutional rate of PONV and shorten the stay in the PACU.

Apfel and colleagues concluded that risk scores can help decrease the incidence of nausea and vomiting by helping the clinical practitioner predict which patients are at high risk for PONV.^41^ They concluded that PONV prophylaxis was more cost-effective for patients who were at higher risk of PONV as determined by the presence of more than two Apfel PONV risk factors.

Frenzel and colleagues^42^ suggested that ongoing collection and reporting of individual clinical performance data helped improve practice behaviour. Retrospectively reviewing 23,279 anaesthetics for PONV guideline compliance, these researchers compared: (i) the effects of using continuing medical education (CME) alone; (ii) CME with a single report of provider compliance and (iii) ongoing provider performance feedback of compliance data without further CME. Compliance was defined as a patient with one risk factor for PONV receiving at least one antiemetic, those with two risk factors receiving at least two antiemetics and those with three risk factors receiving at least three antiemetics. The greatest improvement in guideline compliance occurred with ongoing personal performance feedback.

Kooij and colleagues investigated the effect of an electronic decision support system to enable patient-specific automated reminders.^43^ Use of this system significantly improved the adherence to the prescription of antiemetic prophylaxis in patients at high risk of PONV. However, the authors stated that although mandatory data entry of PONV risk factors improved the identification of patients at high risk, after deactivating the decision support system, the effect on adherence to guidelines disappeared completely.

To help decrease the incidence of PONV in a hospital, a prevention plan can be implemented using PONV risk assessments. In a busy ambulatory surgery practice, symptoms such as nausea may be missed and undetected. Controversy may exist in some practices on whether to give a single antiemetic to nearly all patients as a universal approach, even though these patients have a low risk for PONV.

Kranke and colleagues discussed the pros and cons of a ‘risk-adapted strategy’ compared with a ‘universal multimodal approach’ for PONV prophylaxis.^44^ These researchers state that rather than using a ‘risk-adapted strategy’, a more liberal multimodal prevention strategy minimises the risk in moderate- to high-risk patients who receive suboptimal prophylaxis. A universal approach also minimises the risk to low-risk patients who receive a single treatment that is not effective.

Dewinter and colleagues evaluated a simplified algorithm for PONV prevention.^45^ Female patients received triple antiemetic prophylaxis using dexamethasone, ondansetron and a target-controlled infusion of propofol or droperidol. Male patients received double prophylaxis with dexamethasone and ondansetron. Their results revealed a significantly lower PONV incidence after implementation of the algorithm.

## Conclusions

Adult and paediatric outpatients and inpatients may experience nausea and vomiting because of specific patient-, surgery- and anaesthesia-related risk factors. Children and adults have different aetiologies and incidences for PONV, PDNV or POV. Although specific patient- and surgery-related risk factors can often not be modified, anaesthesia- and medication-related factors may be minimised or even eliminated; TIVA and regional anaesthesia techniques help in this regard. In the perioperative period, nitrous oxide gas, volatile anaesthetics and opioids are common anaesthesia-related predisposing factors. The PONV risk assessments can help decrease the incidence of PONV at a hospital level. The use of PONV protocols and algorithms, electronic medical record reminders regarding postoperative instructions, and feedback to anaesthesia providers can help standardise the approach to prevention and treatment, thereby helping to improve patients' care.

## MCQs

The associated MCQs (to support CME/CPD activity) will be accessible at www.bjaed.org/cme/home by subscribers to *BJA Education*.

## Declaration of interests

The author declares that they have no conflict of interest.
